# Does Shoot Apical Meristem Function as the Germline in Safeguarding Against Excess of Mutations?

**DOI:** 10.3389/fpls.2021.707740

**Published:** 2021-08-05

**Authors:** Agata Burian

**Affiliations:** Institute of Biology, Biotechnology and Environmental Protection, Faculty of Natural Sciences, University of Silesia in Katowice, Katowice, Poland

**Keywords:** germline, somatic mutation, cell division, stem cell, shoot apical meristem

## Abstract

A genetic continuity of living organisms relies on the germline which is a specialized cell lineage producing gametes. Essential in the germline functioning is the protection of genetic information that is subjected to spontaneous mutations. Due to indeterminate growth, late specification of the germline, and unique longevity, plants are expected to accumulate somatic mutations during their lifetime that leads to decrease in individual and population fitness. However, protective mechanisms, similar to those in animals, exist in plant shoot apical meristem (SAM) allowing plants to reduce the accumulation and transmission of mutations. This review describes cellular- and tissue-level mechanisms related to spatio-temporal distribution of cell divisions, organization of stem cell lineages, and cell fate specification to argue that the SAM functions analogous to animal germline.

## Introduction

Each living organism inevitably accumulates mutations due to errors in DNA replication, activity of transposable elements, free radicals, or UV radiation. If not lethal, a mutation is passed on to the descendant cells *via* mitotic cell divisions and generates a clone of mutated cells (Gill et al., 1995). Even though the vast majority of mutations are selectively neutral or only slightly deleterious, they will accumulate with time and eventually decrease an individual’s fitness, presumably causing aging, cancer, and other diseases in case of animals and humans (Erickson, 2014; Vijg, 2014). However, the mutations will not be transmitted to the next generation unless they occur in progenitors of gametes.

Sexually reproducing animals separate a dedicated cell lineage (germline) that gives rise to gametes responsible for genetic continuity of the species. The most prominent feature of animal germline is early specification and separation from somatic lineages already during embryogenesis, which combined with a low mitotic activity, effectively reduces the number of mutations resulting from DNA replication errors (Extavour, 2007). Therefore, functioning of the germline is closely associated with the protection of the genetic material that is transmitted to next generations.

In contrast to animals, the germline in plants is specified late during post-embryonic development (e.g., Berger and Twell, 2011; Grossniklaus, 2011). This may be not risky for a short-lived plant, such as Arabidopsis, however, in long-lived species, late germline specification and continuous mitotic activity could be expected to lead to a high number of cell divisions per generation, the accumulation of mutations, and ultimately “mutational meltdown” (Lynch et al., 1993). Yet, some plants can live and reproduce for several thousands of years (Lanner, 2002; Munne-Bosch, 2018).

This review addresses the question how plants protect their genetic material from mutations occurring during prolonged lifetime *via* mechanisms acting at cellular and tissue levels. First, key features of animal germline and mechanisms are described that reduce the risk of heritable mutations. Then, it is argued that the similar protective mechanisms exist in plants, although they extend beyond the germline *sensu stricto*, and involve the system of continuously generated shoot apical meristems (SAMs). Thus, in this sense, the SAM is functionally analogous to animal germline.

## Germline and Mutation Rate in Animals

**FIGURE 1:**
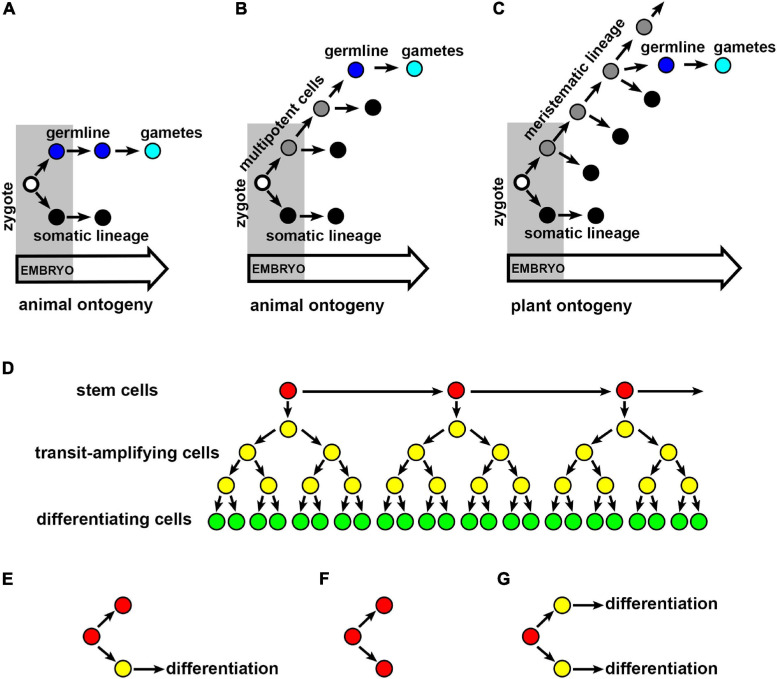
Cell lineages minimizing the risk of heritable mutations. Establishment of the germline and somatic lineages in animals **(A,B)** and plants **(C)**. **(A)** In most of vertebrates (e.g., mouse and chicken) and ecdysozoans (e.g., *Drosophila* and *Caenorhabditis elegans*) the germline (blue) producing gametes (cyan) is specified and separated from somatic lineages (black) during embryogenesis. **(B)** In other animals (e.g., flatworms, cnidarians, or sponges), the germline is specified during post-embryonic development from multipotent lineages (gray) that produce also somatic lineages. **(C)** In plants, the germline is specified during post-embryonic development from meristematic cell lineages (gray), that produces also somatic lineages. Empty circle, zygote; black circle, somatic lineage; blue circle, germline; cyan circle, gametes; gray circle, multipotent or meristematic lineage. **(D)** Hierarchical organization of stem cell lineage. Slowly dividing stem cells (red) produce descendant stem cells and faster dividing transit-amplifying cells (yellow) that eventually enter a differentiation pathway (green). **(E–G)** The fate of stem cell descendants. **(E)** Stem cell descendants after the asymmetric division acquire a different fate: the stem cell (red) and the cell that ultimately differentiate (yellow). Stem cell descendants after the symmetric division acquire the same fate: either both become stem cells **(F)**, or both cells ultimately differentiate **(G)**.

One of the most recognizable features of the animal germline is its early specification (Figure 1A; Saffman and Lasko, 1999; Extavour and Akam, 2003; Strome and Updike, 2015). During the specification, precursors of gametes called primordial germ cells (PGCs) are established that are distinguished from somatic cells by their characteristic histology and molecular markers (Saffman and Lasko, 1999; Extavour and Akam, 2003). After the specification, PGCs are separated from somatic cells, in the sense, that their mitotic activity is reduced, and they do not respond to factors promoting somatic differentiation often being transcriptionally quiescent (Strome and Updike, 2015; Swartz and Wessel, 2015).

In many animals, germline specification and separation occur during embryogenesis. For example, *Drosophila* germline is specified after a series of 10 nuclear divisions in the early syncytial embryo, before cellularization. In *Caenorhabditis elegans* the germline is specified progressively during the first four embryonic divisions, and becomes fully established at the 16 or 24-cell embryo. Early germline specification occurs also in *Xenopus*, that is at 32-cell embryonic stage, or in chicken – at the 300-cell embryonic stage, whereas in the mouse, the germline is specified before or during gastrulation. Therefore, in most of animal model species, the germline is the first established lineage, and its specification occurs before the specification of somatic lineages.

Early specification, however, is not a universal feature of animal germline. For example, differently from most model species, the germline in axolotls is established after gastrulation, thus, after the specification of somatic lineages (Chatfield et al., 2014). The germline can be specified even later, for example, after embryogenesis in annelids or echinoderms, or throughout the adulthood in some flatworms, cnidarians, or sponges (Buss, 1983; Extavour and Akam, 2003; Johnson and Alberio, 2015). In these cases, the germline develops from multipotent stem cells, that give rise to both the germline and somatic cells (Figure 1B; Agata et al., 2006; Juliano and Wessel, 2010). However, these multipotent cells, for example in sea urchin, are separated from other cells already in the embryo, and at least initially remain mitotically quiescent (Juliano et al., 2010). Clearly, the timing of germline specification and separation is not fixed in animals, and it ranges from different stages of embryogenesis to the adulthood.

Early germline specification and separation has an advantage of reducing the number of cell divisions which is expected to protect from the accumulation of somatic mutations (Extavour, 2007). Indeed, typically the mutation rate (defined in this review as the number of mutations per generation) is significantly lower in the germline than in somatic lineages (Lynch, 2010; Milholland et al., 2017; Whittle and Extavour, 2017). Moreover, a difference in germline mutation rate exists between females and males that can be explained by the different number of cell divisions. In mammal females, there is a relatively small number of cell divisions preceding the production of the ovum, which does not increase with age, because all cell divisions are completed before the birth. In mammal males, however, sperm cells are continuously produced during reproductive life, thus, the number of cell divisions increases with the age. For example, in female mice there are 25 germ-cell divisions compared to 62 divisions in males (Drost and Lee, 1995). This difference is even higher in humans, where the number of germ-cell divisions is about 31 in females, while for a 20-year old male this number is already 150, and further increases by more than five times for a 50-year old male (Crow, 2000). Accordingly, the mutation rate in males is much higher than in females, and it increases rapidly with the individual age (Drake et al., 1998; Crow, 2000; Campbell and Eichler, 2013). Interestingly, the difference in germline mitotic activity between sexes is not conserved in animals. In Drosophila, numbers of germ-cell divisions for females and males are similar (about 35–36 divisions), as is the mutation rate (Drost and Lee, 1995).

These data support the idea that there is a relationship between the number of cell divisions and the mutation rate. Therefore, reduced mitotic activity can contribute to the protection of the germline from somatic mutations. However, given the risk of mutation accumulation in the germline where the number of cell divisions is higher, for example, due to late separation, alternative cellular- and tissue-level mechanisms have evolved to minimize the mutation rate (molecular mechanisms have been reviewed, e.g., Strome and Updike, 2015; Raz and Yamashita, 2021).

The organization of the germline, as other types of stem cell lineages in animals, is hierarchical. This means that the lineage consists of slowly dividing self-renewing stem cells, that give rise to faster dividing transit-amplifying cells (undifferentiated cells in a transient state between “stemness” and differentiation), that ultimately produce differentiating cells, e.g., gametes (Figure 1D; Kay, 1965; Cairns, 1975). Consequently, the lineage with relatively few stem cell divisions can generate numerous differentiating cells, the number of which depends on divisions in transit-amplifying cells. This hierarchical lineage organization can limit the accumulation of mutations in the germline. Low mitotic activity of stem cells reduces the probability of replication errors and resulting mutations, which is particularly important, because any mutation that occurs in a stem cell is prone be fixed (Pepper et al., 2007; Derenyi and Szollosi, 2017). Mutations in transit-amplifying cells, even if they are more likely to occur due to higher mitotic activity, never reach fixation and are lost from the cell lineage during cell differentiation.

Moreover, the fate of stem cells is not fully predictable. Although, a stem cell often gives rise to one descendant which retains stem cell fate and the other descendant which differentiates (asymmetric division) (Figure 1E), sometimes both the descendants can acquire the same fate (symmetric division): either of the stem cell or of differentiating cell (Figures 1F,G; Morrison and Kimble, 2006). Indeed, male germline stem cells can stochastically lose their stem cell fate by symmetric cell division, and be replaced by neighboring transit-amplifying cells (Klein and Simons, 2011; Stine and Matunis, 2013). This means that stem cells are not permanent, which have an important consequence in the fate of mutations. Namely, in a cell lineage with permanent stem cells (where at least one stem cell descendant retains stem cell fate), a mutation occurring in the stem cell will indefinitely propagate to all descendants. However, in the case of impermanent stem cells (where both stem cell descendants lose stem cell fate), the mutation is more likely to be lost by stochastic elimination of mutated stem cell followed by its differentiation (Shahriyari and Komarova, 2013; McHale and Lander, 2014).

Therefore, regardless of the diversity in the germline specification and separation, several protective mechanisms, such as low mitotic activity, hierarchical lineage organization, or impermanency of stem cells, have been developed in animal germline to reduce the risk of mutations, restrict fixation of mutations and their accumulation in the germline.

## Germline in Plants

In contrast to animals, in which organ formation is usually completed after embryogenesis, plants generate organs throughout their entire lifetime. The formation of above-ground organs occurs at the SAM containing a population of potentially immortal stem cells that give rise not only to somatic cells, but also gametes (Figure 1C). Other unique feature of plants is the presence of diploid (sporophyte) and haploid (gametophyte) phases of the life cycle (Walbot and Evans, 2003). A cell lineage committed to producing gametes (the germline *sensu stricto*) is specified late in sporophyte development. However, whether the plant germline initiates with spore mother cell formation (Grossniklaus, 2011; She and Baroux, 2014), or with the mature gametophyte (Berger and Twell, 2011), is still a matter of debate. It is also unclear when exactly plant germline is separated from somatic lineages (Lanfear, 2018). In this review, evidences are presented for extending the narrow meaning of germline in plants. In agreement with the concept of functional plant germline first proposed by Romberger et al. (1993), it is argued that SAM functions analogous to animal germline, in that it limits the accumulation and transmission of somatic mutations to next individuals and generations.

In *Arabidopsis*, the SAM is specified in the 16-cell embryo and subsequently forms between cotyledons (Capron et al., 2009). From then on, all above-ground organs derive from the SAM. During the vegetative phase of development, the SAM produces either leaves (organs with determinate growth) or indeterminate axillary meristems. These meristems give rise to lateral shoots and form SAMs, which, again, produce determinate organs and meristems (Domagalska and Leyser, 2011). During the reproductive phase, the SAM produces flower meristems that are homologous to axillary meristems (Long and Barton, 2000; Kwiatkowska, 2006). The flower meristem generates sexual organs where ultimately gametes are formed, which is preceded by development of haploid gametophytes (Feng et al., 2013). Thus, the process of meristem formation is continuous, and consequently, lineages of meristematic cells exist in plants from the embryo to gametes, even though they are not separated from somatic lineages.

**FIGURE 2:**
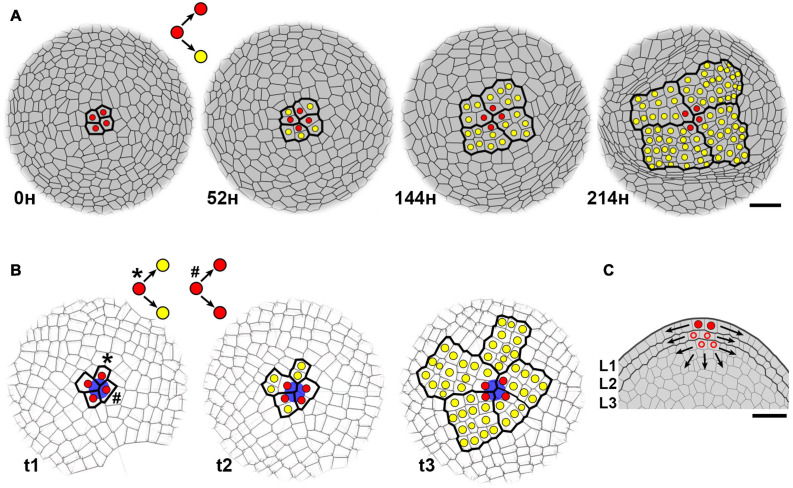
Stochastic and semi-permanent behavior of plant stem cells. **(A)** Dynamics of stem cells at the surface of vegetative SAM in Arabidopsis (top view, based on the figure 4, Burian et al., 2016). Stem cells were identified at L1 (the outermost cell layer of tunica) by tracing of cell lineages based on time-lapse imaging with laser confocal microscopy. A stem cell (red) divides asymmetrically producing the descendant cell that maintains its position at SAM center and stem cell fate, and the cell that loses stem cell fate by its displacement to the periphery, and becomes transit-amplifying cell (yellow) undergoing series of cell divisions. Note, that the same set of four stem cells is functioning for at least 9 days. Scale bar, 20 μm. **(B)** Computer simulation of stochastic stem cell behavior (modified, based on the Video S4, Kucypera et al., 2017). In Kucypera et al. (2017), stem cells were defined by a stable point corresponding to the geometric center of SAM surface. Here, stem cells (red) were defined at t1 by a stable positional information marked by a blue circle. The simulation shows that although these stem cells generally divide asymmetrically, symmetrical divisions can also occur leading to rearrangement of stem cells. Due to cell displacement, both descendants of the stem cell (indicated by an asterisk at t1) lose stem cell fate at t2, and undergo series of cell divisions (yellow). Consequently, the cell clone is displaced from the SAM center at t3. In contrast, both descendants of the other stem cell (indicated by a hash at t1) keep stem cell fate at t2 as they maintain the position at the center. **(C)** Plausible stem cells below SAM surface. At longitudinal section across the SAM (from **A**), thicker lines indicate outer cell layers of the tunica (L1 and L2) and inner corpus (L3). Arrows indicate the direction of cell displacement due to oriented cell divisions. Each tunica layer and the corpus contain their own sets of stem cells. Red circle, stem cell of the L1 (identified at **A**), empty circle, plausible stem cells of L2 and L3. Stem cells of L1 give rise to epidermis, L2 – subepidermal tissues, and gametes, L3 – internal tissues. Scale bar, 20 μm.

The functioning of the SAM depends on activity of stem cells localized at SAM center. The stem cell can be defined as a cell that self-renews and generates differentiating cells (Heidstra and Sabatini, 2014; Slack, 2018). However, there has been a confusion in the plant literature about which cells in the SAM should be named stem cells (Laux, 2003; Kuhlemeier, 2017). In *Arabidopsis*, CLAVATA3, which is expressed in approximately 10–20 cells at SAM surface, is often used as a marker for stem cell identity due to its role in the regulation of stem cell maintenance (Fletcher et al., 1999; Brand et al., 2002). In contrast, observations of clonal sectors at shoots of plant chimeras, or identification of cell clones at single time-point SAM images revealed 2–4 stem cells (called initials or apical initials in classical botanical terminology) in many vascular plants (Stewart and Dermen, 1970; Christianson, 1986; Gola and Jernstedt, 2011; Zagórska-Marek and Turzańska, 2014; Conway and Drinnan, 2017). In Arabidopsis and tomato, tracing of cell lineages based on time-lapse imaging provides a direct method to identify 3–4 stem cells at the SAM surface (Figure 2A; Burian et al., 2016). Therefore, only a subset of CLAVATA3-expressing cells meets the functional criteria of stem cells, that is, self-renewal and generation of differentiating cells.

Similar to animals, the organization of stem cell lineage at the SAM is hierarchical. Namely, descendants of stem cells either retain stem cell fate at the SAM center, or lose this fate and undergo several cell divisions at SAM periphery, before they will give rise to a new organ, e.g., a leaf (Figure 2A; Heidstra and Sabatini, 2014). As these cells are in transition between stem cells and differentiating cells, they are equivalent to animal transit-amplifying cells. At the time of leaf initiation, few of these transit-amplifying cells are arrested in cell divisions and locate at the boundary between a leaf primordium and the SAM (future axil), where they persist in a quiescent state until they give rise to the axillary meristem (Burian et al., 2016; Shi et al., 2016). This quiescent state can last weeks, months, or even years in some trees (Garrison, 1955; Fink, 1984; Meier et al., 2012). Thus, rather than being generated *de novo* from differentiated cells in leaf axil, axillary meristem originates directly from undifferentiated cells of the SAM, in agreement with the “detached meristem” hypothesis (Romberger, 1963; Grbić and Bleecker, 2000; McSteen and Hake, 2001; Greb et al., 2003; Alvarez et al., 2006).

## Mitotic Activity in Plant Meristems

Since the germline *sensu stricto* is specified late in plant ontogeny, the high number of cell divisions from the zygote to gametes (or per generation) is expected, especially in large long-lived plants. Although the estimation of this number is still a major challenge, the already existing evidence suggests that this number cannot be simply extrapolated from plant age or size. Instead, given the continuous mode of meristem formation, per-generation number of cell divisions depends on the mitotic activity of stem cells, and on the number of cell divisions separating axillary meristem from the SAM. The cell divisions accompanying the formation of sexual organs and those directly preceding the specification of the germline, are not considered here, because their number is fixed for a given plant and is not expected to increase during prolonged growth, in contrast to the number of cell divisions in the SAM.

Like in animals, a common feature of plant stem cells is their low mitotic activity (Laufs et al., 1998; Reddy et al., 2004; Kwiatkowska, 2008). In particular, a long cell cycle of 40 days or more has been estimated for trees as compared to 3–12 days in annual plants (Stewart and Dermen, 1970; Lyndon, 1990; Romberger et al., 1993). Importantly, due to hierarchical organization of stem cell lineage, the mitotic activity in stem cells is not proportional to the size of a plant body. For example, stem cell mitotic activity is not correlated with the number of lateral organs (Watson et al., 2016). Instead, the amount of new organs depends on the activity of transit-amplifying cells at SAM periphery, where new organs are initiated. Also, shoot elongation *per se* does not depend on the mitotic activity of stem cells, as it is a result of subapical growth activity in the region comprising internodes between leaves (Romberger, 1963; Maksymowych et al., 1985). Finally, the per-generation number of cell divisions can be further decreased by early detachment of axillary meristem. The number of cell divisions separating the SAM and axillary meristem is relatively low and depends on the size of SAM, rather than on post-meristematic growth of the shoot (Romberger et al., 1993; Burian et al., 2016).

Altogether, for example, about 100 stem cell divisions have been estimated per generation in a 50-year-old tree (Romberger et al., 1993), 120 divisions in a tree with 10^6^ terminal branches (Burian et al., 2016), or 135 divisions in 76-m high spruce (Hanlon et al., 2019). By contrast, 34–50 cell divisions are estimated for annuals, like Arabidopsis or maize (Otto and Walbot, 1990; Watson et al., 2016). Clearly, the number of cell divisions per generation does not increase proportionally to plant growth or lifetime. Given the relationship between the number of cell divisions and the mutation rate, similar tendency is predicted also in the case of the mutation rate.

Indeed, recent genetic analyses show that even long-lived plants achieving considerable sizes do not accumulate as many somatic mutations per generation that could be expected from their extended lifetime and growth (Schmid-Siegert et al., 2017; Plomion et al., 2018; Hanlon et al., 2019; Wang et al., 2019; Hofmeister et al., 2020; Orr et al., 2020). However, the estimation of mutation rate needs further studies, because other analyses (Klekowski and Godfrey, 1989; Ally et al., 2010; Bobiwash et al., 2013; Schoen and Schultz, 2019) predict higher mutation rate. Nonetheless, plants like trees can live for centuries without (or with only weak) physiological signs of aging, that would result from the accumulation of somatic mutations (Lanner and Connor, 2001; Mencuccini et al., 2014; Wang et al., 2020). Thus, reduced mitotic activity in stem cells may contribute to low mutation rate in long-lived plants. Interestingly in this context, there is also no correlation between body size, longevity, and a cancer risk in animals (Caulin and Maley, 2011).

## Stochastic and Semi-Permanent Behavior of Plant Stem Cells

Although the mutation rate can be limited by reduced mitotic activity in stem cells, indeterminate growth inevitably increases the risk of mutations in plant germline. Further protective mechanisms are related with behavior of stem cells.

Botanists have known for a long time, that stem cells do not function permanently in most of vascular plants (Ruth et al., 1985; Klekowski, 1988; Green et al., 1991; Gola and Jernstedt, 2011; Zagórska-Marek and Turzańska, 2014; Conway and Drinnan, 2017; Jill Harrison, 2017). The same set of 3–4 stem cells can persist at the SAM center and function for quite a long time, as long as each cell divides asymmetrically, so that one descendant cell retains stem cell fate, whereas the other loses this fate, becomes transit-amplifying cell, and after series of cell divisions, ultimately differentiates (Figure 2A). For example, tracing of cell lineages in Arabidopsis revealed that the same set of stem cells can function for 7–9 days (Burian et al., 2016), while in other annuals, the same stem cells function through both vegetative and reproductive phase of development (Lyndon, 1998). However, like in animals, plant stem cells can be also stochastically lost and replaced by neighboring cells meaning that they do not function permanently. How then the fate of plant stem cells is regulated? Stem cell fate is controlled by a positional information provided by the WUS-CLV feedback, hormone action (reviewed in Heidstra and Sabatini, 2014; Fuchs and Lohmann, 2020; Lopes et al., 2021), and perhaps also by mechanical signals (Kierzkowski et al., 2012). Thus, plant stem cells are better viewed as “*the temporary occupants of a permanent office*” (Newman, 1965). Once a stem cell leaves the “*office*,” it loses its fate. Accordingly, if positional information is stable at SAM center, the loss or acquirement of stem cell fate depends on cell displacement which is determined by the direction of growth and the orientation of cell division plane. Cell growth is slow and isotropic (uniform in all directions) at SAM center, and the orientation of anticlinal cell division planes is random (Kwiatkowska, 2004; Louveaux et al., 2016). In a consequence, eventually both descendants of a stem cell can be displaced from the center and lose the stem cell fate, while a new stem cell can be recruited from descendants of the other neighboring stem cell (Figure 2B). However, to understand the role of growth and cell division patterns in determination of stem cell fate, further studies are needed.

Summarizing, since plant stem cells persist at the SAM center through prolonged time, but ultimately are stochastically lost, they can be described as semi-permanent. Such a behavior arises from the position-dependent control of stem cell fate and cell displacement that decides whether a cell retains or loses its fate. Therefore, the fate of stem cells is to some extent stochastic, as long as the growth at meristem center is isotropic and the orientation of cell divisions is random.

## The Fate of Somatic Mutations

A mutation, if not eliminated by DNA repair system, cell cycle arrest, or cell death (Bray and West, 2005; Fulcher and Sablowski, 2009; Hu et al., 2016), will be propagated in dividing cells. Thus, cell divisions are not only a major source of mutations, they also allow the mutation to spread within individual organism and to the offspring. Here, only neutral mutations are considered because they cannot be eliminated by the natural selection (mechanisms underlying the selection of non-neutral mutations at different levels of plant organization have been described, e.g., Whitham and Slobodchikoff, 1981; Walbot and Evans, 2003; Schoen and Schultz, 2019).

The highest chance that a mutation will be fixed at the SAM and propagate infinitely, is when the mutation occurs in stem cells. Mutation fate, however, will depend on the number and permanency of stem cells. For example, the mutation in a single permanent stem cell, which occurs at the SAM of mosses or some ferns (Philipson, 1990; White and Turner, 1995; Gola and Banasiak, 2016; Jill Harrison, 2017), will be immediately fixed (Klekowski, 2003). In contrast, larger number of stem cells and their semi-permanency will favor stochastic loss of mutations. The rate of spontaneous mutations estimated for Arabidopsis is 7⋅10^–9^ base substitutions per site per generation, which gives approximately 1 mutation per genome per generation (Ossowski et al., 2010; Yang et al., 2015). Given 3–4 semi-permanent stem cells, it is unlikely, that mutations occur in all of them at the same time. Also, if the mutation occurs in one stem cell, there is a low chance that this mutated cell will keep the position at SAM center and replace all other stem cells (Burian et al., 2016). Instead, it is more likely, that the mutated stem cell will be ultimately displaced from the center, so that the mutation will be lost by differentiation, or it will be transmitted to axillary meristem. However, the mutation can be fixed only in those axillary meristems that are generated within a mutated sector occupying 1/3 or 1/4 of the circumference of the shoot (i.e., the sector generated by one stem cell, that width depends on the number of stem cells; Figure 2A). Thus, continuous formation of axillary meristems that ultimately give rise to new branches, prevents the uniform distribution of somatic mutations throughout the shoot, but instead, it may lead to hierarchically extending mutated sectors, such as recently found in oak tree (Schmid-Siegert et al., 2017).

The fate of mutations depends also on the SAM structure. In dicots, SAM structure is layered, meaning that the displacement of stem cells and their fate is partially restricted by prevalence of anticlinal cell divisions (Figure 2C; Lyndon, 1998). In a consequence, such structure enhances the retention of mutated cell clones, for example, in the form of stable periclinal chimeras (Klekowski, 1988; Frank and Chitwood, 2016). In monocots and gymnosperms, however, stem cell division plane is not restricted, or is restricted to a lesser extent (Stewart and Dermen, 1979; Lyndon, 1998; Conway and Drinnan, 2017), thus, the elimination of mutated stem cells and their clones can be more efficient (Klekowski, 1988).

Altogether, the fate of somatic mutations depends on stem cell behavior and cellular processes such as cell divisions or cell growth. Permanency of stem cells and layered SAM structure, promote the fixation of mutations in a plant, while larger number of stem cells, their semi-permanency, and non-layered SAM structure allow for stochastic loss of mutations.

## Conclusion

Despite fundamental differences in animal and plant development, similar cellular- and tissue-level mechanisms exist to reduce the amount of heritable mutations. These mechanisms include low mitotic activity and hierarchical organization of stem cell lineage, which minimizes the risk of mutations, and stochastic behavior of stem cells which facilitates the loss of mutations. In plants, a body plan is not determined during embryogenesis, but develops progressively during entire lifetime, and cell lineages in the SAM give rise to both somatic cells and gametes. Nonetheless, these cell lineages are continuous from the embryo to gametes, and transmit genetic information to next generations or to clonally propagated individuals. Thus, given the presence of protective mechanisms and cell lineage continuity, the SAM is functionally analogous to animal germline. Because somatic mutations not only decrease individual and population fitness, but also enable evolution, the SAM participates in the protection of genetic information, but at the same time allows the adaptation to changing environment and to rapidly evolving pathogens or insects (Whitham and Slobodchikoff, 1981; Simberloff and Leppanen, 2019).

It is still an open question whether similar protection against the accumulation of somatic mutations occurs in flower organs. Recent advances in live imaging techniques, which enable to follow cellular events during the specification of spore mother cells or during the gametophyte development, will likely help to reveal mechanisms of such protection in future (Prusicki et al., 2019; Valuchova et al., 2020; Hernandez-Lagana et al., 2021; Susaki et al., 2021; Vijayan et al., 2021).

## Author Contributions

The author had made a substantial, direct and intellectual contribution to the work, and approved it for publication.

## Conflict of Interest

The author declares that the research was conducted in the absence of any commercial or financial relationships that could be construed as a potential conflict of interest.

## Publisher’s Note

All claims expressed in this article are solely those of the authors and do not necessarily represent those of their affiliated organizations, or those of the publisher, the editors and the reviewers. Any product that may be evaluated in this article, or claim that may be made by its manufacturer, is not guaranteed or endorsed by the publisher.
